# Comparing the Clinical Characteristics and Mortality of Residential and Non-Residential Older People with COVID-19: Retrospective Observational Study

**DOI:** 10.3390/ijerph19010483

**Published:** 2022-01-02

**Authors:** Francesc X. Marin-Gomez, Jacobo Mendioroz-Peña, Miguel-Angel Mayer, Leonardo Méndez-Boo, Núria Mora, Eduardo Hermosilla, Ermengol Coma, Josep-Maria Vilaseca, Angela Leis, Manolo Medina, Queralt Miró Catalina, Josep Vidal-Alaball

**Affiliations:** 1 Health Promotion in Rural Areas Research Group, Gerència Territorial de la Catalunya Central, Institut Català de la Salut, 08772 St. Fruitós de Bages, Spain; xmarin.cc.ics@gencat.cat (F.X.M.-G.); jmendioroz.cc.ics@gencat.cat (J.M.-P.); jvidal.cc.ics@gencat.cat (J.V.-A.); 2 Unitat de Suport a la Recerca de la Catalunya Central, Fundació Institut Universitari per a la Recerca a l’Atenció Primària de Salut Jordi Gol i Gurina, 08772 St. Fruitós de Bages, Spain; qmiro.cc.ics@gencat.cat; 3 Faculty of Medicine, University of Vic-Central University of Catalonia (UVIC-UCC), 08500 Vic, Spain; josepmaria.vilaseca@umedicina.cat; 4 COVID-19 Response Unit, Department of Health, Generalitat de Catalunya, 08005 Barcelona, Spain; 5 Research Programme on Biomedical Informatics (GRIB), Hospital del Mar Medical Research Institute (IMIM), Universitat Pompeu Fabra, 08003 Barcelona, Spain; angela.leis@upf.edu; 6 Sistemes d’Informació dels Serveis d’Atenció Primària (SISAP), Institut Català de la Salut (ICS), 08007 Barcelona, Spain; lmendezboo@gencat.cat (L.M.-B.); nmora@idiapjgol.info (N.M.); ehermosilla@idiapjgol.info (E.H.); ecomaredon@gencat.cat (E.C.); mmedinap@gencat.cat (M.M.); 7 Fundació Institut Universitari per a la Recerca a l’Atenció Primària de Salut Jordi Gol i Gurina (IDIAPJGol), 08007 Barcelona, Spain

**Keywords:** COVID-19, epidemiology, multimorbidity, nursing home, institutionalisation, mortality

## Abstract

Nursing homes have accounted for a significant part of SARS-CoV-2 mortality, causing great social alarm. Using data collected from electronic medical records of 1,319,839 institutionalised and non-institutionalised persons ≥ 65 years, the present study investigated the epidemiology and differential characteristics between these two population groups. Our results showed that the form of presentation of the epidemic outbreak, as well as some risk factors, are different among the elderly institutionalised population with respect to those who are not. In addition to a twenty-fold increase in the rate of adjusted mortality among institutionalised individuals, the peak incidence was delayed by approximately three weeks. Having dementia was shown to be a risk factor for death, and, unlike the non-institutionalised group, neither obesity nor age were shown to be significantly associated with the risk of death among the institutionalised. These differential characteristics should be able to guide the actions to be taken by the health administration in the event of a similar infectious situation among institutionalised elderly people.

## 1. Introduction

Nursing homes accommodate elderly people with a sufficient degree of autonomy for the activities of daily living and who require a substitute home environment (accommodation, food, shelter, cohabitation and personal support) or elderly people who do not have this autonomy and therefore need constant supervision and the replacement of the home by an environment that is adapted to their degree of dependence [1]. Estimates indicate that a significant proportion of population mortality is concentrated in these centres [2].

At the social level, mortality in residential facilities is higher in areas with poorer socio-economic conditions, and people with lower socio-economic status, living alone or without children, are more likely to die in a nursing home [3,4]. People living in urban areas appear to be more likely to end up in nursing homes than patients in rural areas, although this does not appear to have an impact on median survival [5]. The place of death depends substantially on socio-demographic determinants, such as household characteristics and living conditions, as well as regional factors [6,7]. The causes of high mortality in this population are very diverse: they vary in relation to sex and increase with age at admission, the degree of dependence to perform activities of daily living, the number of recent hospitalisations, the burden of existing comorbidities (especially cardio-cerebrovascular, neurodegenerative, pulmonary or renal diseases), the presence of severe psychotic symptoms, the total pharmacological treatments prescribed, a worse nutritional state of the person or the lack of physical activity [4,5,8,9,10].

Compared to the general population, a significantly higher increase in mortality has been demonstrated for COVID-19, associated with a greater number of chronic diseases, greater multimorbidity and the mean number of medications taken (11.7 vs. 8.0) [11]. This increase in mortality with age, especially in people over 65 years of age [12], is probably due to the fact that severe forms of the disease are more common, especially in very old people [12,13]. Although the transmission of the subclinical form in this age group appears to be infrequent, it is not common [14]. The high transmissibility of this virus means that its dissemination can be much greater than in other spaces, and can even be increased by the limitations of these centres, as they do not have the resources and staff of a hospital or primary care centre in infection control and prevention nor the means of individual protection for staff members working in these facilities [12,15].

For all these reasons, there is currently significant social alarm regarding mortality in these centres as a result of SARS-CoV-2 infection, and the media has made them one of the main focuses of attention during the weeks of the pandemic. Although studies have been carried out to establish which factors were predictive of mortality in residential care homes [16], no comparisons were made of the impact these factors had internally or externally.

From a clinical point of view, it is of the utmost importance to identify predictors of mortality in nursing homes. A recent article determined that the predictors of mortality in this population are: male, aged over 80 years, hospitalisation for COVID-19, cardiovascular disease, chronic kidney disease and dementia [17]. Another study carried out in a group of homes in Catalonia on residents with a positive reverse transcriptase polymerase chain reaction (RT-PCR) showed that, in addition to certain symptoms due to infection, the comorbidities significantly associated with death in homes were exclusively the presence of dementia and liver disease [16].

The objectives of the study are, firstly, to analyse the mortality associated with SARS-CoV-2 infection during the first wave of the pandemic among institutionalised elderly persons (IOP) ≥ 65 years and compare it with those of the rest of the population of the same age (non-IOP). Secondly, to determine the influence that various factors may have on mortality in IOP ≥ 65 years. Our hypothesis is that there are factors, such as certain diseases or health conditions, that are associated with higher mortality from COVID, among those over 65 years of age, and that these factors vary if we differentiate the cases according to whether they were institutionalised or not.

## 2. Materials and Methods

### 2.1. Study Design and Data Sources

The entire study has been described in accordance with the recommendations proposed by strengthening the reporting of observational studies in epidemiology (STROBE).

A retrospective cohort study included all individuals ≥ 65 years (1,319,839 persons), whether IOP or non-IOP, alive at the start of the first pandemic wave [18], assigned to all primary care centres in Catalonia, Spain. For the study, retrospective data were collected on COVID-19 cases, coinciding with the first wave period, occurring between 1 March and 30 April 2020 [18], and the study individuals were grouped according to whether or not they resided in a geriatric centre in Catalonia. We excluded those people who had no linkage to the electronic health records (EHR) because they were not assigned to one of the primary care practices contributing to our database (146,723 persons).

For our study, different data sources were used and analysed separately: IOPs were identified from the records of the Primary Care Services Information System (SISAP) in Catalonia, Spain [19,20]. The SISAP contains anonymised primary care electronic medical records of more than 6 million people, covering more than 80% of the Catalan population. It includes diagnoses coded according to the International Classification of Diseases, 10th Revision, Clinical Modification (ICD-10-CM), medication prescriptions, laboratory tests, and sociodemographic and lifestyle information. In addition, the data were linked to the regional central RT-PCR database for SARS-CoV-2, mortality registers and electronic primary care medical records. Data from these databases have been previously validated and used for epidemiological research—refs. [21,22], including some studies on COVID-19 [23,24,25,26].

### 2.2. Baseline Characteristics and Comorbidities

The total number of confirmed and suspected COVID-19 cases was used for the study. Confirmed cases were defined as COVID-19 infections confirmed by laboratory RT-PCR. Suspected cases were defined as those diagnosed with COVID-19 infection, according to the International Classification of Diseases, Tenth Revision, Clinical Modification (ICD-10-CM codes B34.2, B97.21, B97.29, J12.81), either in their medical history or on their death certificate. Sociodemographic data were evaluated at the date of the beginning of the study, referring to: gender, age (in years and age ranges), place of residence (institutionalised in nursing home or not institutionalised), rurality (rural, urban) and the composite socio-economic index (CSI). Rural areas were defined as areas with less than 10,000 inhabitants and a population density of less than 150 inhabitants/km^2^. The CSI was used to assess the socio-economic status [27].

Comorbidities were defined as the presence of a diagnosis code recorded prior to the study start date and still active at the time of COVID-19 diagnosis, for a pre-specified list of diseases based on the previous literature [28,29]. ICD-10-CM codes for each of these conditions are provided in Table 1. Codes related to COVID-19 are provided in Table S1.

Complexity was stratified on the basis of adjusted morbidity groups (AMG) and chronic complex patient (CCP) or advanced chronic disease (ACD) groupings. In AMGs, the risk or complexity strata are generated from the complexity that the grouper assigns to each individual in the population. For this purpose, 4 cut-off points are used based on the 50th, 80th, 95th and 99th percentiles. This allows people to be classified into five levels of complexity according to the cut-off percentile: (1) Initial complexity (healthy stage), with a complexity score up to the 50th percentile of the total population; (2) Low complexity, with a score between the 50th and 80th percentiles; (3) Moderate complexity, with a score between the 80th and 95th percentiles; (4) High complexity, with a score between the 95th and 99th percentiles; (5) Very high complexity, with a score above the 99th percentile [30]. In this way, the population with a level of individual complexity below the 50th percentile is grouped in the lowest risk or complexity stratum. This group represents 50% of the reference population. On the contrary, the individuals of the population whose level of individual complexity is higher than the 99th percentile are grouped in the stratum of greater risk or complexity and represent 1% of the population. When we talk about CCP/ACD we refer to patients with complex health needs (severe disease or multimorbidity, polypharmacy, high resource utilisation and concurrent social risk), who may have special needs and a more difficult management. In Catalonia, structured proposals have been established to identify these patients as CCP or, in the presence of a limited life expectancy, as patients with ACD [31,32,33].

In Spain, the restriction policies and the blockade were not imposed until the government declared a state of emergency on 14 March 2020, when the first wave had already begun. In the nursing homes, during this period, age and comorbidity profiles were added to factors such as the intensity of the pandemic outbreak, lack of molecular diagnostic tools and protective equipment, and lack of staff training. On the other hand, the role of the primary care in the supervision of residences, until then marginal, had to be provided from public centres, when the first wave was already advanced and within a very short period of time.

### 2.3. Outcomes

The outcome of the study was death due to COVID-19 among the participating cases from the start date of the study until 28 days after the end of the study. Deaths due to COVID-19 infection were identified using a list of ICD-10-MC diagnosis codes recorded in the patients’ medical record (Table 1), and factors that can influence mortality in individuals ≥ 65 years old, like age, gender, rurality, economical status and complexity or comorbidities were also obtained. The date of death was obtained from the linked regional mortality data.

### 2.4. Statistical Methods

The results are presented as number of cases (n) and percentages (%) for categorical variables and mean (standard deviation) in the continuous variables. To assess the effect of the main confounding variables on mortality (gender, age, and adjusted morbidity group (AMG)), stratified analyses were performed (gender (male, female), age (65–74, 75–84 and ≥85 years)). The incidence of COVID-19 cases and their mortality were estimated by adjusting the mortality rate by age, and the WHO reference population was used [34] to standardise the results.

To assess the association between COVID-19 diagnoses and death events, a Cox regression model was used. Model fit by gender, age or place of residence was estimated. To test the robustness of the model stratification method, concordance indices were estimated and an ANOVA analysis was performed.

All analyses were performed with the statistical program R and RStudio: Integrated Development for R. RStudio, Inc., Boston, MA, USA. The significance level was set at 5% and all confidence intervals at 95%.

## 3. Results

### 3.1. Characteristics of the Sample

From all the subjects at the start of the study (1,466,562), we excluded those subjects who were not correctly linked in all the medical records used (146,723) [non-electronic health records linkage available/non-EHR linkage] and from whom it was not possible to obtain the clinical study variables.

**Figure 1:**
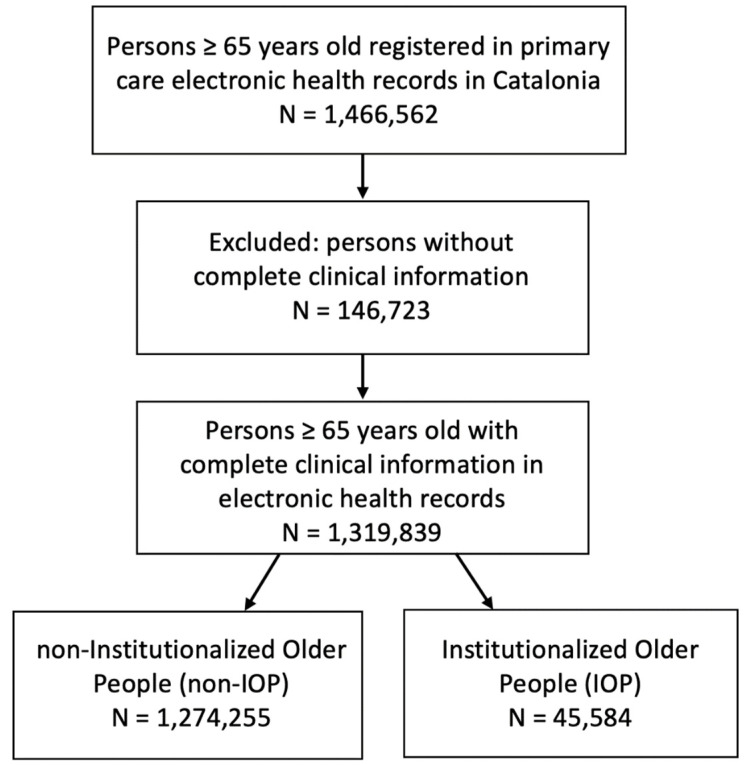
Flow chart with inclusion and exclusion criteria.

The cohort studied included 1,319,550 individuals ≥ 65 years old assigned to primary care centres in Catalonia during the first wave of the pandemic, between March and April 2020 (Figure 1).

The mean age of the cohort was 75.9 (SD 7.95), with the majority being between 65 and 74 years of age. Women were a total of 754,921 (57.2%), with a mean age of 76.6 (SD 8.25), while in men it was 75.0 (SD 7.43). Among men there is a higher prevalence of COPD (14.5% compared to 3.99%), ischaemic heart disease (12.9% compared to 4.93%) and cancer (25.0% compared to 17.1%), while among women there is a higher prevalence of obesity (31.8% compared to 27.0%), dementia (7.06% compared to 4.12%) and osteoarthritis (46.2% compared to 27.8%).

The cohort was grouped according to whether they were elderly people living in long-term institutions or “Institutionalised older people” (IOP). The IOP group was 45,584 (3.45%), while the non-IOP group was 1,274,255 (96.5%). The mean age of the IOPs (86.4 (SD 7.35)) was significantly higher (*p* < 0.001) than among non-IOPs (75.5 (SD 7.71)). The majority of IOPs were in the age range of over 84 years 29,976/45,584 (65.8%), while the majority of non-IOPs were under 75 years 659,690/1,274,255 (51.8%), showing a significant difference (*p* < 0.001) in relation to the frequency of cases in these age groups. Females were the majority in both groups, being significantly higher in the case of the IOP group, in which there were 33,716 (74.0%). Few differences were observed in terms of the rural or urban origin of the two populations.

The analysis of comorbidities in the two groups showed a greater number of complex (CCP) or palliative (ACD) patients among the IOP, with the number of CCP among the IOP being 21,656 (47.5%) compared to 108,605 (8.52%) in the non-IOP (*p* < 0.001). In the case of advanced or palliative patients (ACD), there were 5347 (11.7%) among the IOP, compared to 14,883 (1.17%) in the non-IOP (*p* < 0.001).

In the majority of the comorbidities studied, a higher prevalence was observed among the IOP, except in the case of obesity, with 9024 (19.8%) cases among the IOP, while in the non-IOP it was 38,3707 (30.1%) and in some non-viral liver diseases, in which the double frequency was observed among the non-IOP, 96,766 (7.59%), with respect to the IOP, 1572 (3.45%). The most prevalent comorbidities among IOPs are dementia, 10 times more frequent than among non-IOPs; chronic kidney disease, 10% more frequent; cardiovascular disease, twice as frequent; and, finally, osteoarthritis, 7% more frequent among IOPs than non-IOPs (Table 2).

When we analyse the joint presence of several comorbidities, we observe that the majority of non-IOPs (52.6%) present two or fewer comorbidities, while in the case of IOPs the majority (75.5%) present three or more comorbidities, the differences being even more marked in the case of patients presenting four or more morbidities (residents with 55.1% compared to non-residents with 28.8%).

### 3.2. COVID-19 Cases

The incidence of COVID-19 cases among the IOP was higher, with 28.8% (13,140/45,584), while among the non-IOP population we found only 1.88% (23,970/1,274,255). This difference is observed both in cases confirmed by RT-PCR (22.1% in IOPs vs. 1.05% in non-IOPs) and in cases diagnosed by the clinic (6.71% in IOPs vs. 0.83% in non-IOPs). A total of 35.8% of all RT-PCRs performed (11,160/31,138) were conducted on IOPs (these being only 3.45% (45,584/1,319,839) of the population), resulting positive in 90.4% of cases (10,083/11,160), compared to 67.1% in non-IOPs (13,405/19,978).

COVID-19 cases included by clinical diagnosis (without confirmatory RT-PCR) constitute 44.1% (10,565/23,970) of the non-IOP COVID-19 cases and 23.3% (3057/13,144) of the IOP cases.

#### Cumulative Incidence Curve of COVID-19 Cases

As can be seen in the graph (Figure 1) of weekly cumulative incidence of COVID-19 cases during the first wave, the peak of weekly cumulative cases occurred on 29 March 2020.

**Figure 2:**
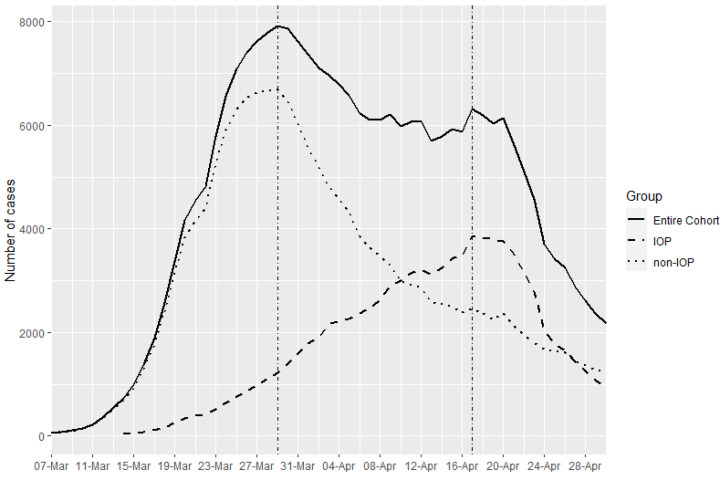
Weekly cumulative incidence curve of COVID-19 cases by place of residence.

If we draw the curve of cases according to whether they were IOP we can see that there is a time lag with respect to the time when the peak of cases occurs. Outside the institutions, the peak weekly cumulative incidence occurs on 29 March, and in the case of residences it occurs on 17 April, which is 19 days (approximately 3 weeks) later (Figure 2).

### 3.3. Mortality Due to COVID-19

As much as 43.3% (4212/9726) of deaths among COVID-19 cases occurred among IOPs. This number of deaths represents a 32.1% (4212/13,140) COVID-19 case fatality rate in the IOP population and 9.24% (4212/45,584) of the total IOP population. Deaths among COVID-19 cases in non-IOPs accounted for 23% (5514/23,970), being 0.41% of the total non-IOP study population.

The mean age of death among the COVID-19 cases in the population aged ≥65 years during the study period was 84.6 years (SD: 8.03), being significantly higher among the IOP population, 87.8 (SD: 6.86), and among non-residents, 82.2 (SD: 7.99). By age group, the age group with the greatest differences was that over 84 years of age (73.3% compared to 42.6%) with an OR of 7.04 (95% CI 5.97–8.36).

While, in general, a higher number of deaths was observed among females (51.8% in women, compared to 48.2% in men; *p* < 0.001), when stratifying according to whether they were IOP, it was observed that the trend was accentuated among IOPs, the number of deaths among IOP women being significantly higher (*p* < 0.001) (64.6% in women vs. 35.4% in men), with an OR = 2.33 (2.33–2.75).

The mean ICS of the COVD-19 deaths of the study was 43.61 (SD: 15.6), being slightly lower in the case of the IOP group, with 41.3 (SD: 14.5), compared to 45.3 (SD: 16.2) in non-IOPs.

The incidence of COVID-19 deaths was significantly higher among the urban population (79.9% in urban areas compared to 20.1% in rural areas), with little difference when comparing the IOP (urban 76.8%) and non-IOP (urban 82.1%) groups, OR = 0.72 (0.64–0.80). The mortality among cases confirmed by RT-PCR in the IOP population was lower (43.6%) than among those diagnosed clinically (56.4%), behaving inversely in the non-IOP population, where deaths among those confirmed by RT-PCR was 62.1% compared to those diagnosed clinically, with 37.9% (OR = 2.12, 95% CI 1.95–2.31).

The analysis according to the complexity of the participants shows that among the IOP population there was a higher percentage of deaths among the CCP (47.9% vs. 27.1%) or ACD (14.8% vs. 5.09%) patients, OR= 2.47 (2.26–2.69) in the CCP and OR = 3.23 (2.78–3.77) in the case of the ACD.

The deaths with comorbidities are more numerous in the non-IOP population, except for cerebrovascular disease (10.9% in IOP vs. 9.30% in non-IOP), OR = 1.19 (1.04–1.37), dementia (51.2% in IOP vs. 13.9% in non-IOP), OR = 6.52 (5.90–7.22), and osteoarthritis (44.5% in IOP vs. 42.8% in non-residents), OR = 1.06 (0.98–1.15); although this difference was only significant in the case of dementia (*p* < 0.001) and cerebrovascular disease (*p* = 0.012).

If we group comorbidities (none, 1, 2, 3 or >3), we observe that in the non-IOP population, COVID-19-related deaths are more frequent when two or fewer morbidities are associated, while from three or more associated morbidities the frequency increases significantly in nursing homes. The frequency of deaths among the IOP population with three morbidities is 19.4% compared to 18% in the non-residents, OR 1.49 (1.25–1.78), (*p* < 0.001); while when residents present four or more associated morbidities, their frequency increases to 58.1%, compared to the 54.2% of non-residents, with an OR = 1.48 (1.27–1.74) and a *p* < 0.001 (Table 3).

#### 3.3.1. Weekly Mortality Rate by Place of Residence

The age-adjusted weekly mortality rate for COVID-19 in IOP was 62.6 (95% CI: 62.0–63.2) per 1000 individuals, while this mortality rate among non-IOP was 3.35 (95% CI: 3.34–3.55).

An estimate of the mortality rate by age, sex and type of case (confirmed by RT-PCR or clinical diagnosis), stratified according to whether or not it is an IOP, shows that mortality increases in all age groups, but that the increase is greater as the age range increases (Table 4).

**Figure 3:**
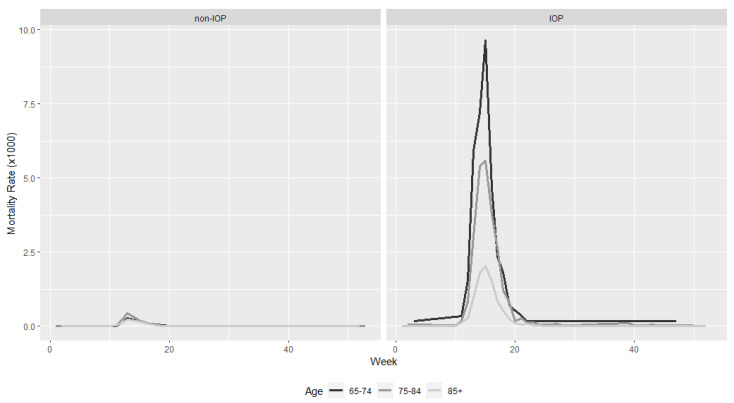
Age-adjusted mortality curves of COVID-19 cases by place of residence.

The age range with the greatest difference, in terms of age-standardised mortality rates, is that of 65–74, where it goes from being 1.04 (95% CI: 1.03–1.04) per thousand in non-IOPs to 33.9 (95% CI: 32.9–35.1) per thousand in the IOP population (Figure 3). This difference is also observed in terms of the gender of the participants, with males having a higher mortality rate, but this difference is more relevant in the IOP population, with an adjusted rate between 65 and 74 years of age of 41.3 (95%CI: 39.4–43.2) in IOP men with respect to 1.53 (95% CI: 1.52–1.53) among non-IOPs.

#### 3.3.2. Model of Overall Mortality Risk among COVID-19 Cases, According to the Place of Residence (sHR: Stratified Hazard Ratio)

The proportional hazards model or Cox regression, adjusted for place of residence, showed that age appears to be significantly associated (*p* < 0.001) with a greater probability of death during the pandemic within the ≥65 years population (sHR 1.01, 95% CI 1.00–1.02) and specifically in non-IOPs (HR 1.02, 95% CI 1.01–1.03). In contrast, residence in an urban setting is also associated with increased risk for the entire cohort (sHR 1.09, 95% CI 1.03–1.15) and among IOPs (HR 1.12, 95% CI 1.03–1.21), but not significantly so for non-IOPs. However, male gender accounts for a higher probability of death for the entire cohort (sHR 1.24, 95% CI 1.18–1.31), both among IOPs (HR 1.19, 95% CI 1.11–1.29) and non-IOPs (HR 1.27, 95% CI 1.19–1.35). The level of complexity of the patient as measured by the five levels of the AMG classification is also a higher risk. Thus, as the AMG level of the cohort moves from level 1 to 5, so does the HR, which is 11.53 (95% CI 10.8–12.2) for level 1 and 43.5 (95% CI 6.05–312.7) at level 5. The risk weight of the last level only falls on the IOPs (HR 29.5, 95% CI 4.02–217.4), given that there is no level 5 case among the non-IOPs.

The analysis of comorbidities and their impact on the probability of death from COVID-19 during the first wave of the pandemic shows that obesity is associated with an increased probability of death (sHR 1.08, 95% CI 1.03–1.15) and that this increased probability is only found among non-IOPs (HR 1.10, 95% CI 1.03–1.19). In the case of dementia, we also observed an increased likelihood of death overall, in the whole cohort (HR 1.21, 95% CI 1.12–1.28), but in this case it is associated with an isolated increased likelihood among IOPs (HR 1.32, 95% CI 1.22–1.44).

The Cox model applied on the entire cohort was stratified by IOP, given that the ANOVA analysis between the stratified and non-stratified model showed clearly significant differences (χ^2^ = 11,476; *p* ≤ 0.001). Thus, the concordance of the stratified model used was 0.82 (SE = 0.002). The model used among the IOP population obtained a concordance of 0.76 (SE = 0.004), while in non-IOP it was 0.85 (SE = 0.003). (Table 5).

Several models were made adjusting for age and gender, but the model that obtained the highest concordance index was the one adjusted for place of residence (IOP), with a concordance of 0.82 (SE 0.002), showing itself as the model that best agrees with the observed survival results.

## 4. Discussion

The results of the study show the existence of a clearly differentiated behaviour of the incidence of cases during the first wave of COVID-19 in Catalonia. The cumulative incidence of cases in the non-IOP population follows a steeper curve, starting at the beginning of the study period (1 March 2020) and reaching its peak weekly cumulative incidence of cases on 29 March (4 weeks after the start). In contrast, in the IOP population, the peak weekly cumulative incidence occurs on 17 April (7 weeks after the start of the epidemic), with a less pronounced increase in the number of cases. Thus, it can be observed that the difference between maximum peaks of cumulative incidence of cases is 19 days (approximately 3 weeks). Despite this difference in cumulative incidence growth, the date of detection of the first cases is similar. The first case in non-IOPs is reported on 1 March while in IOPs it is reported on 3 March (two days later). This differentiated behaviour in the cumulative incidence can be explained by the organisation of the clusters of institutions. The occurrence of cases or an outbreak in one institution limits the transmission to the number of possible cases among its residents, but does not lead to an increased risk to a distant institution. The system of physical isolation between institutions would seem to be a possible cause of the slower overall contagion curve among IOPs. Such a delay could be seen as an opportunity or advantage to prevent, more effectively than in the non-IOP population, the slow but inevitable spread of cases [35,36,37].

The findings also corroborate the conclusions of other studies [38,39] in relation to the lethality of SARS-CoV-2 infection. It appears that once the first cases occur, the mortality rate among IOPs is much higher than among non-IOPs. In the two study groups it is observed that as we increase the age range of the individuals, the mortality rate also increases, as it also does among males. On the other hand, it is worth noting that unlike the non-IOP population, among IOPs a higher mortality rate is detected among those clinically diagnosed (without confirmatory RT-PCR), and this fact could corroborate the thesis of studies indicating that the spread of cases among IOPs was more extensive than previously estimated using RT-PCR tests alone [40].

The factor that best explained the increased likelihood of dying from COVID-19 was the level of complexity of the AMGs. The fact of presenting some level of complexity according to the grouping or case-mix used seems to be associated with a higher probability of death, and this probability increases as we increase the level of complexity studied. A previous study has already established such an association, revealing a 7% increase in the proportion of COVID-19 deaths among residents with complex conditions [41]. When we analyse the other demographic factors, we see that the male gender, as noted in previous studies, is also associated with a higher probability of death among COVID-19 cases, whether IOP or not [42]. A better understanding of the influence of gender on mortality in the study cohort would be of great interest, so it would be interesting to be able to carry out an additional study dividing the general cohort by gender. However, the differences appear when we analyse the results according to age and the population’s area of residence, given that among the non-IOP population the increase in age is significantly associated with a greater probability of death, while this is not true in the case of the IOP; and the opposite occurs in the case of belonging to an urban area, which is significantly associated with a greater probability of death only among the IOP. This association had already been detected in previous studies [43]. The data from our study confirm this fact, which is not significant among non-IOPs. The analysis of comorbidities shows a higher risk among non-IOPs for individuals with obesity and diabetes, while among IOPs the comorbidity that is associated with a higher probability of death is clearly dementia. Studies conducted before the pandemic by COVID-19 already showed that people with dementia were more vulnerable individuals, whose daily survival often depends on other caregivers. A previous meta-analysis has already shown that patients with dementia have twice the risk of death associated with pneumonia [44]. A recent meta-analysis revealed that dementia appears to be associated with an increased risk of mortality from COVID-19 infection and proposes, as a cause, that most patients with dementia, because of their advanced age, already have other comorbid medical conditions that likely increase the severity and mortality of COVID-19 infections; in addition, these may present with atypical symptoms that may mask the severity of the individual’s life situation [45]. Given the clear association with poor prognosis among IOPs with dementia, they should be treated with special care and follow-up to minimise the exposure to the virus. Professionals attending institutions with the elderly should perform a close surveillance of dementia patients with suspected COVID-19 for early diagnosis and treatment to prevent mortality from COVID-19. Dementia should be considered an important risk factor in future COVID-19 risk stratification models, especially in nursing homes or institutions where elderly people reside.

The results of this study may enable management and decision-making bodies to plan, prioritise and develop more precise actions to protect vulnerable elderly people in the event of new outbreaks of SARS-CoV-2 infection. On the other hand, in a more indirect way, they could also contribute to the design of new models of health care in residential centres, at a time when this area is undergoing a profound reconsideration.

To carry out an analysis of the association between the characteristics of the study cohort and dying from COVID-19 (inside and outside institutions for the elderly) we used records of PCR diagnostic test results (reference test) but also of ICD-10-CM diagnoses made by clinicians, in the absence of confirmatory PCR testing. This could have resulted in the inclusion of misclassified cases, as other similar diseases such as influenza or respiratory conditions could have been diagnosed as COVID-19 in the context of the current pandemic. However, prior validation of medical history records exists for a variety of conditions, including influenza [46,47]. Specifically, COVID-19 cases as defined here (based on PCR, but also based on clinical diagnoses) have been used to provide information for pandemic management at all levels, reaching down to the health professional level with patient lists updated daily from primary care information systems, which has allowed the monitoring of cases but also the review and correction of possible misclassification errors. On the other hand, the use of a hard outcome variable such as mortality helps to control for potential recording problems [23].

The study has different limitations, among which we can highlight that it has been carried out among patients assigned to residential groups in their electronic medical records at the time of analysis, without taking into account possible displacements or changes of place of residence that may have occurred during the study period. On the other hand, as mentioned in the discussion, institutionalised individuals are grouped into clusters of more or fewer individuals, sometimes with very different characteristics and environments, that make it difficult to generalise the results.

An important limiting factor of the study is the fact that for the diagnosis of coronavirus infection, some diagnoses of clinical suspicion that had not been confirmed by previous RT-PCR were included in our study. The fact that the first pandemic wave will cause an unexpected and very high number of COVID-19 deaths in IOPs meant that some cases of COVID-19 that resulted in the death of the subject were diagnosed a posteriori, without the possibility of prior confirmatory testing. As seen in the results, the majority of cases that ended in the death of the IOP are based on clinical diagnoses, and to have considered only those confirmed by PCR would still have introduced a greater bias or error in the conclusions.

The findings of the study could help to improve the management and prioritisation of the available resources for the protection of vulnerable populations in the future, saving economic and social costs through an appropriate and coordinated intervention to reduce the high mortality caused by an epidemic such as SARS-CoV-2, both nationally and internationally.

## 5. Conclusions

Our risk model, based on demographic data and various morbidities, identified risk groups among the population ≥65 years, and specifically among elderly patients institutionalised in nursing homes, who are particularly prone to increased mortality. The clinical complexity of individuals, in general and with dementia, among the IOP, was an important factor in explaining the increased risk of mortality. IOPs with dementia were more likely to die from COVID-19 infection in a nursing home than residents without dementia.

## Figures and Tables

**Table 1:** Comorbidities of interest and related code lists.

Comorbidity	ICD10-CM Codes
Hypertension	I10, I11.0, I11.9, I12.0, I12.9, I13.0, I13.10, I13.2, I15.0, I15.1, I15.2, I15.8, I15.9
Type 1 Diabetes Mellitus	E10.10, E10.29, E10.311, E10.359, E10.39, E10.49, E10.59, E10.621, E10.65, E10.69, E10.8, E10.9
Type 2 Diabetes Mellitus	E11.01, E11.21, E11.22, E11.29, E11.311, E11.39, E11.40, E11.43, E11.49, E11.51, E11.59, E11.610. E11.621, E11.638, E11.641, E11.649, E11.65, E11.69, E11.8, E11.9, E13.10, E13.29, E13.39, E13.49, E13.59, E13.641, E13.69, E13.8, E13.9
Chronic obstructive pulmonary disease (COPD)	J43.0, J43.1, J43.2, J43.8, J43.9, J44, J44.0, J44.1, J44.9
Ischaemic heart disease	I20, I20.0, I20.8, I20.9, I24.0, I24.1, I24.8, I24.9, I25, I25.10, I25.2, I25.41, 125.5, I25.6, I25.89, I25.9, I70.90, I21, I21.0, I21.01, I21.02, I21.09, I21.1, I21.11, I21.19, I21.2, I21.29, I21.3, I21.4, I22.0, I22.1, I22.2, I22.8, I22.9, I23.0, I23.1, I23.2, I23.3, I23.4, I23.5, I23.6, I23.8
Cerebrovascular disease	G45.0, G45.1, G45.2, G45.8, G45.9, G46.0, G46.1, G46.2, G46.3, G46.4, G46.5, G46.6, G46.7, G46.9, I63.00, I63.10, I63.20, I63.30, I63.40, I63.50, I63.6, I63.8, I63.9, I67.82, I67.9, I69.30, I69.320 I69.398, I69.80, I69.998 I61.0, I61.1, I61.2, I61.3, I61.4, I61.5, I61.6, I61.8, I61.9, I61.10
Heart failure	I50.1, I50.20, I50.30, I50.32, I50.9
Atrial fibrillation	I48.0. I48.1, I48.2, I48.91
Liver failure	K70.0, K70.10, K70.2, K70.30, K70.9, K73.0, K73.1, K73.2, K73.8, K73.9, K75.0, K75.2, K75.3, K75.4, K75.89, k75.9, K76.5, K76.7, K76.9
Type B Hepatitis	B16.0, B16.1, B16.2, B16.9,
Type C Hepatitis	B17.10, B18.2
Cancer (all except non-melanoma skin cancer)	C00-C97 (except C44), D00-D09
Chronic Kidney Disease	I12.0, I13.0, N03.9, N18.1, N18.2, N18.4, N18.5, N18.6, N18.9, N19, N28.9, N99.0, P96.0, Z94.0
Obesity	E66.01, E66.09, E66.1, E66.2, E66.8, E66.9

**Table 2:** Comparison of participant characteristics according to whether they were institutionalised (IOP) or not.

	Entire Cohort N = 1,319,839 N (%)	Non-IOP N = 1,274,255 N (%)	IOP N = 45,584 N (%)	*p*-Value
Age (in years), mean (SD)	75.9 (7.95)	75.5 (7.71)	86.4 (7.35)	<0.001
Age group (at baseline)				<0.001
65–74 years	663,341 (50.3)	659,690 (51.8)	3651 (8.01)	
75–84 years	430,020 (32.6)	418,063 (32.8)	11,957 (26.2)	
≥85 years	226,478 (17.2)	196,502 (15.4)	29,976 (65.8)	
Gender				<0.001
Female	754,921 (57.2)	721,205 (56.6)	33,716 (74.0)	
Male	564,918 (42.8)	553,050 (43.4)	11,868 (26.0)	
Rurality				<0.001
Rural	271,847 (25.0)	259,269 (24.7)	12,578 (33.1)	
Urban	814,547 (75.0)	789,072 (75.3)	25,475 (66.9)	
CSI, mean (SD)	45.2 (15.5)	45.3 (15.6)	42.1 (13.8)	<0.001
CSI Levels				<0.001
<25	110,381 (10.2)	105,390 (10.1)	4991 (13.1)	
25–49	605,789 (55.8)	582,095 (55.5)	23,694 (62.3)	
50–74	329,262 (30.3)	320,542 (30.6)	8720 (22.9)	
>75	40,962 (3.77)	40,314 (3.85)	648 (1.70)	
AMG_complexity Levels				<0.001
Level 0	1,262,621 (95.7)	1,229,661 (96.5)	32,960 (72.3)	
Level 1	39,025 (2.96)	29,964 (2.35)	9061 (19.9)	
Level 2	15,716 (1.19)	12,532 (0.98)	3184 (6.98)	
Level 3	2308 (0.17)	1949 (0.15)	359 (0.79)	
Level 4	163 (0.01)	144 (0.01)	19 (0.04)	
Level 5	6 (0.00)	5 (0.00)	1 (0.00)	
CCP	130,261 (9.87)	108,605 (8.52)	21,656 (47.5)	<0.001
ACD	20,230 (1.53)	14,883 (1.17)	5347 (11.7)	<0.001
Medical conditions (at baseline)				
Hypertension	824,012 (62.4)	791,399 (62.1)	32,613 (71.5)	<0.001
Diabetes mellitus	323,150 (24.5)	310,573 (24.4)	12,577 (27.6)	<0.001
COPD	112,149 (8.5)	108,206 (8.49)	3943 (8.65)	0.237
Ischaemic heart disease	110,223 (8.35)	106,139 (8.33)	4084 (8.96)	<0.001
Cerebrovascular disease	74,986 (5.68)	70,037 (5.50)	4949 (10.9)	<0.001
Congestive heart failure	80,693 (6.11)	74,648 (5.86)	6045 (13.3)	<0.001
Atrial fibrillation	147,705 (11.2)	139,402 (10.9)	8303 (18.2)	<0.001
Valvular disease	86,984 (6.59)	84,088 (6.60)	2896 (6.35)	0.039
Liver failure	98,338 (7.45)	96,766 (7.59)	1572 (3.45)	<0.001
Type B Hepatitis	4628 (0.35)	4483 (0.35)	145 (0.32)	0.248
Type C Hepatitis	11,318 (0.86)	10,743 (0.84)	575 (1.26)	<0.001
Cancer	270,160 (20.5)	261,455 (20.5)	8705 (19.1)	<0.001
Chronic kidney disease	226,811 (17.2)	214,490 (16.8)	12,321 (27.0)	<0.001
Obesity	392,731 (29.8)	383,707 (30.1)	9024 (19.8)	<0.001
Dementia	76,550 (5.80)	56,104 (4.40)	20,446 (44.9)	<0.001
Osteoarthritis	505,726 (38.3)	485,175 (38.1)	20,551 (45.1)	<0.001
Num. of Comorbidities				<0.001
0	164,305 (12.4)	162,761 (12.8)	1544 (3.39)	
1	251,173 (19.0)	245,949 (19.3)	5224 (11.5)	
2	287,447 (21.8)	278,163 (21.8)	9284 (20.4)	
3	256,654 (19.4)	245,988 (19.3)	10,666 (23.4)	
≥4	360,260 (27.3)	341,394 (26.8)	18,866 (41.4)	
COVID Cases				
Confirmed by RT-PCR	23,488 (1.78)	13,405 (1.05)	10,083 (22.1)	<0.001
Clinical diagnosis	13,622 (1.03)	10,565 (0.83)	3057 (6.71)	<0.001
Deaths	9726 (0.74)	5514 (0.43)	4212 (9.24)	<0.001

CSI: Composed socioeconomic index; AMG: adjusted morbidity group; CCP: Chronic complex patient; ACD: advanced chronic disease; COPD: chronic obstructive pulmonary disease.

**Table 3:** COVID-19 deaths among detected cases comparing institutionalised and non-institutionalised individuals.

	Total Cases N = 37,110 N (%)	Deaths N = 9726 N (%)	*p*-Value	Non-IOP N = 5514 N (%)	IOP N = 4212 N (%)	OR (CI95)	*p*-Value
Age (in years), mean (SD)	80.7 (9.15)	84.6 (8.07)	<0.001	82.1 (8.04)	87.8 (6.86)	1.11 [1.10;1.11]	<0.001
Age group (at baseline)			<0.001				<0.001
65–74 years	11,088 (29.9)	1288 (13.2)		1091 (19.8)	197 (4.68)	Ref.	
75–84 years	11,510 (31.0)	3009 (30.9)		2090 (37.9)	919 (21.8)	2.43 [2.06;2.89]	
≥85 years	14,512 (39.1)	5429 (55.8)		2333 (42.3)	3096 (73.5)	7.34 [6.27;8.65]	
Gender			<0.001				<0.001
Female	21,972 (59.2)	5092 (52.4)		2345 (42.5)	2747 (65.2)	2.53 [2.33;2.75]	
Male	15,138 (40.8)	4634 (47.6)		3169 (57.5)	1465 (34.8)	Ref.	
Rurality			0.080				<0.001
Rural	6536 (21.2)	1677 (20.5)		828 (18.0)	849 (23.8)	1.43 [1.28;1.59]	
Urban	24,253 (78.8)	6486 (79.5)		3775 (82.0)	2711 (76.2)	Ref.	
CSI, mean (SD)	44.3 (15.7)	43.5 (15.6)	<0.001	45.3 (16.1)	41.2 (14.4)	0.98 [0.98;0.99]	<0.001
CSI Levels			<0.001				<0.001
<25	3434 (11.2)	941 (11.5)		463 (10.1)	478 (13.4)	Ref.	
25–49	17,683 (57.4)	4861 (59.5)		2606 (56.6)	2255 (63.3)	0.84 [0.73;0.96]	
50–74	8465 (27.5)	2079 (25.5)		1316 (28.6)	763 (21.4)	0.56 [0.48;0.66]	
>75	1207 (3.92)	282 (3.45)		218 (4.74)	64 (1.80)	0.29 [0.21;0.39]	
AMG complexity Levels			<0.001				0.020
Level 0	26,782 (72.2)	2425 (24.9)		1422 (25.8)	1003 (23.8)		
Level 1	7127 (19.2)	5092 (52.4)		2748 (49.8)	2344 (55.7)		
Level 2	2765 (7.45)	1922 (19.8)		1142 (20.7)	780 (18.5)		
Level 3	405 (1.09)	271 (2.79)		191 (3.46)	80 (1.90)		
Level 4	30 (0.08)	15 (0.15)		11 (0.20)	4 (0.09)		
Level 5	1 (0.00)	1 (0.01)		0 (0.00)	1 (0.02)		
CCP	10,666 (28.7)	3500 (36.0)	<0.001	1494 (27.1)	2006 (47.6)	2.45 [2.25;2.66]	<0.001
ACD	2354 (6.34)	911 (9.37)	<0.001	279 (5.06)	632 (15.0)	3.31 [2.86;3.84]	<0.001
Medical conditions (at baseline)							
Hypertension	25,303 (68.2)	7040 (72.4)	<0.001	4014 (72.8)	3026 (71.8)	0.95 [0.87;1.04]	0.308
Diabetes mellitus	10,635 (28.7)	3154 (32.4)	<0.001	1959 (35.5)	1195 (28.4)	0.72 [0.66;0.78]	<0.001
COPD	4216 (11.4)	1304 (13.4)	<0.001	898 (16.3)	406 (9.64)	0.55 [0.48;0.62]	<0.001
Ischaemic heart disease	4063 (10.9)	1288 (13.2)	<0.001	872 (15.8)	416 (9.88)	0.58 [0.51;0.66]	<0.001
Cerebrovascular disease	3366 (9.07)	976 (10.0)	<0.001	515 (9.34)	461 (10.9)	1.19 [1.04;1.36]	0.010
Congestive heart failure	4428 (11.9)	1488 (15.3)	<0.001	872 (15.8)	616 (14.6)	0.91 [0.82;1.02]	0.113
Atrial fibrillation	6380 (17.2)	1917 (19.7)	<0.001	1165 (21.1)	752 (17.9)	0.81 [0.73;0.90]	<0.001
Valvular disease	2881 (7.76)	823 (8.46)	0.003	556 (10.1)	267 (6.34)	0.60 [0.52;0.70]	<0.001
Liver failure	2527 (6.81)	545 (5.60)	<0.001	417 (7.56)	128 (3.04)	0.38 [0.31;0.47]	<0.001
Type B Hepatitis	141 (0.38)	32 (0.33)	0.393	21 (0.38)	11 (0.26)	0.69 [0.32;1.41]	0.399
Type C Hepatitis	441 (1.19)	129 (1.33)	0.159	72 (1.31)	57 (1.35)	1.04 [0.73;1.47]	0.910
Cancer	8584 (23.1)	2468 (25.4)	<0.001	1653 (30.0)	815 (19.3)	0.56 [0.51;0.62]	<0.001
Chronic kidney disease	8981 (24.2)	2942 (30.2)	<0.001	1751 (31.8)	1191 (28.3)	0.85 [0.78;0.92]	<0.001
Obesity	10,476 (28.2)	2564 (26.4)	<0.001	1808 (32.8)	756 (17.9)	0.45 [0.41;0.49]	<0.001
Dementia	8045 (21.7)	2933 (30.2)	<0.001	766 (13.9)	2167 (51.4)	6.57 [5.96;7.24]	<0.001
Osteoarthritis	16,156 (43.5)	4230 (43.5)	0.929	2357 (42.7)	1873 (44.5)	1.07 [0.99;1.16]	0.094
Number of Medical conditions			<0.001				<0.001
0	2362 (6.36)	367 (3.77)		236 (4.28)	131 (3.11)	Ref.	
1	4968 (13.4)	998 (10.3)		556 (10.1)	442 (10.5)	1.43 [1.12;1.84]	
2	7323 (19.7)	1740 (17.9)		926 (16.8)	814 (19.3)	1.58 [1.25;2.00]	
3	7794 (21.0)	2072 (21.3)		1084 (19.7)	988 (23.5)	1.64 [1.31;2.07]	
≥4		4549 (46.8)		2712 (49.2)	1837 (43.6)	1.22 [0.98;1.53]	
COVID Cases			<0.001				<0.001
Confirmed by RT-PCR	23,488 (63.3)	5505 (56.6)		3489 (63.3)	2016 (47.9)		
Clinical diagnosis	13,622 (36.7)	4221 (43.4)		2025 (36.7)	2196 (52.1)		

CSI: Composed socioeconomic index; AMG: adjusted morbidity group; CCP: Chronic complex patient; ACD: advanced chronic disease; COPD: chronic obstructive pulmonary disease.

**Table 4:** Cumulative weekly mortality rate by age group or gender of COVID-19 cases.

	Non-IOP			IOP		
	Deaths N = 5514	Population N = 1,274,255	MR ^1^ (95% CI)	Deaths N = 4212	Population N = 45,584	MR ^1^ (95% CI)
Age group						
65–74 years	1091	659,690	1.04 (1.03–1.04)	197	3651	33.95 (32.85–35.06)
75–84 years	2090	418,063	1.047 (1.47–1.48)	919	11,957	21.31 (20.93–21.69)
≥85 years	2333	196,502	0.84 (0.84–0.85)	3091	29,976	7.36 (7.28–7.44)
Gender						
Female						
65–74 years	349	354,609	0.62 (0.62–0.62)	78	1832	26.73 (25.51–27.95)
75–84 years	787	238,703	0.97 (0.97–0.98)	495	7971	18.33 (17.92–18.73)
≥85 years	1209	127,839	0.67 (0.67–0.68)	2174	23,797	6.49 (6.4–6.57)
Male						
65–74 years	742	305,053	1.53 (1.52–1.53)	119	1810	41.28 (39.37–43.18)
75–84 years	1303	179,320	2.14 (2.13–2.15)	424	3944	31.72 (30.73–32.71)
≥85 years	1124	68,613	1.16 (1.15–1.17)	922	6059	10.8 (10.53–11.08)
COVID Cases						
RT-PCR						
65–74 years	794	5088	97.97 (95.28–100.6)	113	776	91.42 (84.99–97.85)
75–84 years	1433	4956	85.33 (82.95–87.7)	472	2576	54.07 (51.98–56.16)
≥85 years	1262	3253	29.91 (28.88–30.94)	1431	6567	16.8 (16.39–17.21)
ICD-10-CM						
65–74 years	297	5038	37.01 (35.99–38.03)	84	149	353.9 (297.1–410.7)
75–84 years	657	3209	60.42 (58.33–62.51)	447	687	192.0 (177.6–206.3)
≥85 years	1071	2308	35.78 (34.32–37.24)	1665	2214	57.98 (55.57–60.4)

^1^ Age-standardised mortality rates according to the WHO standard population 2000–2025 [34].

**Table 5:** Results of multivariate risk models of mortality stratified by place of residence.

	Entire Cohort *			Non-IOP			IOP		
	sHR	95% CI	*p*-Value	HR	95% CI	*p*-Value	HR	95% CI	*p*-Value
Age (in years)	1.01	1.00–1.02	<0.001	1.02	1.01–1.03	<0.001	1.00	0.99–1.01	0.295
Age: 75 to 84 years	1.39	1.26–1.53	<0.001	1.23	1.09–1.39	<0.001	1.12	0.92–1.35	0.229
Age: >84 years	1.37	1.18–1.58	0.000	1.09	0.90–1.32	0.361	1.25	0.98–1.59	0.066
Gender: Male	1.24	1.18–1.31	<0.001	1.27	1.19–1.35	<0.001	1.19	1.11–1.29	<0.001
SCI	1.00	0.99–1.00	0.187	1.00	0.99–1.00	0.067	0.99	0.99–1.00	0.548
SCI: 25 to 49	0.92	0.83–1.02	0.138	0.90	0.78–1.04	0.169	0.98	0.85–1.14	0.893
SCI: 50 to 74	0.85	0.73–0.99	0.049	0.80	0.64–0.98	0.039	0.97	0.77–1.24	0.860
SCI: >75	0.83	0.65–1.07	0.161	0.74	0.54–1.03	0.076	1.10	0.71–1.70	0.641
Rurality: Urban	1.09	1.03–1.15	0.001	1.06	0.98–1.15	0.093	1.12	1.03–1.21	0.004
AMG level 1	11.53	10.8–12.2	<0.001	16.93	15.6–18.3	<0.001	6.86	6.31–7.46	<0.001
AMG level 2	12.91	11.9–13.9	<0.001	19.44	17.4–21.6	<0.001	7.68	6.85–8.62	<0.001
AMG level 3	14.33	12.3–16.6	<0.001	22.16	18.3–26.7	<0.001	8.36	6.41–10.9	<0.001
AMG level 4	8.86	5.17–15.1	<0.001	14.05	7.38–26.7	<0.001	4.89	1.79–13.3	0.001
AMG level 5	43.52	6.05–312.7	<0.001	-	-	-	29.55	4.01–217.4	<0.001
CCP	0.90	0.85–0.94	<0.001	0.80	0.75–0.87	<0.001	0.95	0.88–1.03	0.236
ACD	0.68	0.62–0.73	<0.001	0.57	0.49–0.65	<0.001	0.75	0.67–0.83	<0.001
Hypertension	0.97	0.91–1.03	0.400	1.07	0.93–1.10	0.693	0.93	0.84–1.03	0.178
Diabetes mellitus	1.05	0.99–1.11	0.060	1.08	1.00–1.16	0.034	0.99	0.90–1.08	0.846
COPD	0.90	0.84–0.97	0.007	0.91	0.84–1.00	0.059	0.85	0.74–0.96	0.011
Ischaemic heart disease	1.00	0.93–1.08	0.831	1.04	0.95–1.14	0.301	0.94	0.83–1.06	0.339
Cerebrovascular disease	0.96	0.88–1.04	0.330	0.93	0.83–1.03	0.200	1.01	0.89–1.13	0.862
Congestive heart failure	0.90	0.84–0.97	0.008	0.89	0.81–0.98	0.020	0.93	0.83–1.04	0.222
Atrial fibrillation	0.92	0.86–0.98	0.015	0.91	0.84–0.99	0.031	0.93	0.84–1.02	0.159
Valvular disease	0.96	0.88–1.04	0.401	0.95	0.86–1.05	0.376	0.97	0.84–1.12	0.711
Liver failure	0.97	0.88–1.07	0.599	1.03	0.92–1.16	0.503	0.78	0.64–0.96	0.021
Type B Hepatitis	0.97	0.67–1.39	0.870	1.15	0.73–1.82	0.534	0.78	0.43–1.44	0.439
Type C Hepatitis	1.01	0.83–1.22	0.906	1.09	0.84–1.41	0.494	0.97	0.72–1.30	0.871
Cancer	0.95	0.95–1.05	0.089	0.94	0.88–1.01	0.144	0.94	0.85–1.03	0.233
Chronic kidney disease	1.00	0.94–1.06	0.953	0.99	0.92–1.07	0.889	0.99	0.90–1.08	0.898
Obesity	1.09	1.03–1.15	0.002	1.10	1.03–1.19	0.005	1.06	0.96–1.17	0.197
Dementia	1.21	1.14–1.28	<0.001	1.04	0.95–1.15	0.318	1.32	1.22–1.44	<0.001
Osteoarthritis	0.96	0.91–1.01	0.151	0.96	0.89–1.03	0.278	0.99	0.91–1.07	0.830
Comorbidities num.: 1	0.85	0.74–0.98	0.026	0.87	0.72–1.04	0.143	0.67	0.54–0.84	<0.001
Comorbidities num.: 2	0.72	0.62–0.83	<0.001	0.73	0.60–0.88	0.001	0.54	0.42–0.68	<0.001
Comorbidities num.: 3	0.59	0.50–0.70	<0.001	0.60	0.48–0.74	<0.001	0.44	0.34–0.58	<0.001
Comorbidities num.: ≥4	0.54	0.44–0.66	<0.001	0.52	0.40–0.67	<0.001	0.42	0.31–0.58	<0.001

sHR subdistribution hazard ratio, CI confidence interval, y year, na not available, SDI social deprivation index, CCD complex chronic patient, ACD advanced chronic disease, COPD chronic occlusive pulmonary disease, AMG adjusted morbidity group. * Stratified by IOP.

## Data Availability

The datasets used and/or analysed during the current study are available from the corresponding author upon reasonable request.

## Supplementary Materials

The following are available online at https://www.mdpi.com/article/10.3390/ijerph19010483/s1, Table S1. Code lists for EHR attributable to COVID-19 disease.
