# Supplementary material for: Comparing the Clinical Characteristics and Mortality of Residential and Non-Residential Older People with COVID-19: Retrospective Observational Study
Source: Int J Environ Res Public Health. 2022 Jan 2;19(1):483. doi: 10.3390/ijerph19010483 (PMC8744689; doi:10.3390/ijerph19010483)
Supplement: Supplementary file 1 [file ijerph-19-00483-s001.zip › ijerph-1518186-supplementary.pdf]

Supplementary information

Table S1. Code lists for EHR attributable to COVID-19 disease.

ICD-10-CM	Description
B34.2	Coronavirus infection, unspecified
B97.29	Other coronavirus as the cause of diseases classified elsewhere
B97.89	Other viral agents as the cause of diseases classified elsewhere
B34.9	Viral infection, unspecified
B97.21	SARS-associated coronavirus as the cause of diseases classified elsewhere
